# High serum lactate dehydrogenase predicts an unfavorable outcome in Chinese elderly patients with multiple myeloma

**DOI:** 10.18632/oncotarget.16237

**Published:** 2017-03-15

**Authors:** Yan Gu, Ya-Hui Yuan, Ji Xu, Qing-Lin Shi, Xiao-Yan Qu, Rui Guo, Hua Bai, Jia-Dai Xu, Jian-Yong Li, Li-Juan Chen

**Affiliations:** ^1^ Department of Hematology, The First Affiliated Hospital of Nanjing Medical University, Jiangsu Province Hospital, Nanjing, China; ^2^ Department of Oncology, Nanjing Second Affiliated Hospital of Southeast University, Nanjing, China

**Keywords:** LDH, multiple myeloma, R-ISS, outcome

## Abstract

There is significant heterogeneity among multiple myeloma (MM) patients with the survival duration varying greatly from a few months to several years. This study retrospectively analyzed serum lactate dehydrogenase (LDH) in 105 cases of newly diagnosed elderly MM patients to investigate its value for outcome prediction. Serum LDH concentrations were evaluated prior to induction therapy. Prognostic analyses were carried out based on LDH levels and patients’ other clinical data. We also applied the recently proposed Revised International Staging System (R-ISS) to 70 patients with the available data. Of all the patients, elevated serum LDH levels (≥271U/L) were observed in 13.3% (14 out of 105) patients at diagnosis. Compared with normal LDH group, high LDH group had significantly shorter overall survival (OS) (15.5 *vs*. 52.5 months, *p* = 0.002) and median progression free survival (PFS) (12.0 *vs*. 24 months, *p* = 0.030), as well as 2-year OS rate (20% *vs*. 81%, *p* < 0.001) and PFS rate (22% *vs*. 44%, *p* = 0.005). A multivariate analysis identified high LDH as a unique independent adverse prognostic parameter for both OS and PFS. In addition, there were significant differences between R-ISS II and R-ISS III patients in both median OS (52.5 *vs*. 15.5 months, *p* < 0.001) and PFS (23 *vs*. 7.5 months, *p* = 0.004). Furthermore, high LDH was a unique independent adverse indicator for overall response rate (ORR) and early death in elderly MM patients. These results identified LDH as an unfavorable prediction for the outcome of Chinese elderly patients with MM. R-ISS based on LDH is superior to ISS in prognostic assessment.

## INTRODUCTION

Multiple myeloma (MM) is a hematological malignancy characterized by malignant proliferation of abnormally cloned plasma cells that produces monoclonal immunoglobulin, thus resulting in clinical symptoms including infection, anemia, kidney damage and bone lesion [1]. There is significant heterogeneity among MM patients with the survival duration varying greatly from a few months to several years [2].

Glycolysis is disjointed with the citric acid cycle in malignant tumor cells. The production of glucose metabolites is 5~10 times in tumor cells than that in normal tissues, most of which is converted to lactate that increase the release of enzyme to human blood [3]. Among these enzymes, serum lactate dehydrogenase (LDH) is widespreadly distributed in human tissue. LDH is involved in tumor initiation and metabolism. Cancer cells rely on increased glycolysis resulting in increased lactate production in addition to aerobic respiration in the mitochondria, even under oxygen-sufficient conditions. So it has been identified as a sensitive index for hypoxia, anaerobic glycolysis, malignant transformation in cellular metabolism [4]. It has been reported that elevated LDH is correlated with increased disease aggressiveness, high proliferation rate, and the presence of tumor mass [5–7].

Detection of serum LDH level may provide important insights for disease diagnosis, treatment efficacy assessment and prognostic prediction of hematological malignant tumors such as non-Hodgkin lymphoma, chronic lymphocytic leukemia and multiple myeloma [8].

It has been shown that MM is more commonly seen in elderly people with median onset age of 65 years. The incidence of MM increases considerably with age and this disorder rarely occur under the age of 40 years. Despite its frequency, relatively limited information is available regarding the survival and outcome in elderly patients with MM [2,6]. Larocca et al. have reported that improvement in survival is much less pronounced in patients aged 60 to 69 years, and no improvement has been observed in older patients [9]. Given the high incident and the heterogeneity, it's of great importance to investigate the prognostic factors and risk stratification in elderly MM patients to utilize standardized and individualized treatment. This study retrospectively analyzed serum LDH levels in 105 Chinese elderly newly diagnosed multiple myeloma (NDMM) patients, investigating its correlation with other clinical indicators at diagnosis and its value for the outcome prediction, in order to make appropriate individualized assessment of outcome among elderly patients with MM and to design effective and practical risk-adapted therapeutic strategies.

## RESULTS

### Association between LDH level and other clinic characteristics in elderly patients at diagnosis

The median blood platelet count (BPC) was higher in normal LDH group than that in high LDH group (*p* = 0.047), while β2 microglobulin (β2-MG) level was lower (*p* = 0.047). There were no statistical differences between the both groups in patient's other clinical characteristics such as albumin, hemoglobin (HB), C-reactive protein (CRP), ferritin, urea nitrogen, creatinine (sCr), and plasma cells count (PCs) in bone marrow (Table 1).

**Table 1:** Comparison between two groups of patients in clinical characteristic according to serum LDH level at diagnosis

Characteristic	High LDH group	Normal LDH group	*p*
ALB HB BPC β2-MG CRP FER ESR BUN sCr PCs	33.2 (26.6-43.0) 75 (39-118) 119 (13-230) 8.69 (3.08-33.70) 4.15 (3.16-169.0) 641.1 (33.0-7830.0) 100 (15-140) 7.97 (3.52-21.91) 103.9 (38.6-756.8) 15.2 (3.0-84.8)	30.9 (16.3-47.2) 81 (40-136) 150 (13-406) 5.18 (1.37-63.1) 3.79 (3.16-150.0) 328.4 (81.8-1500.0) 105 (5-157) 6.23 (0.90-37.05) 85.2 (35.4-1071.3) 25.8 (0-96.0)	0.067 0.282 0.047 0.047 0.535 0.270 0.515 0.112 0.246 0.097

Abbrevations: ALB, Serum Albumin; HB, Hemoglobin; BPC, Platelet; β 2-MG, β 2-Microglobulin; CRP, C-reactive protein; FER, Ferritin; ESR, Erythrocyte sedimentation rate; BUN, Blood urea nitrogen; sCr, Serum creatinine; PCs, Plasma cells in bone marrow

### High LDH level correlated with inferior OS and PFS

**Figure 1:**
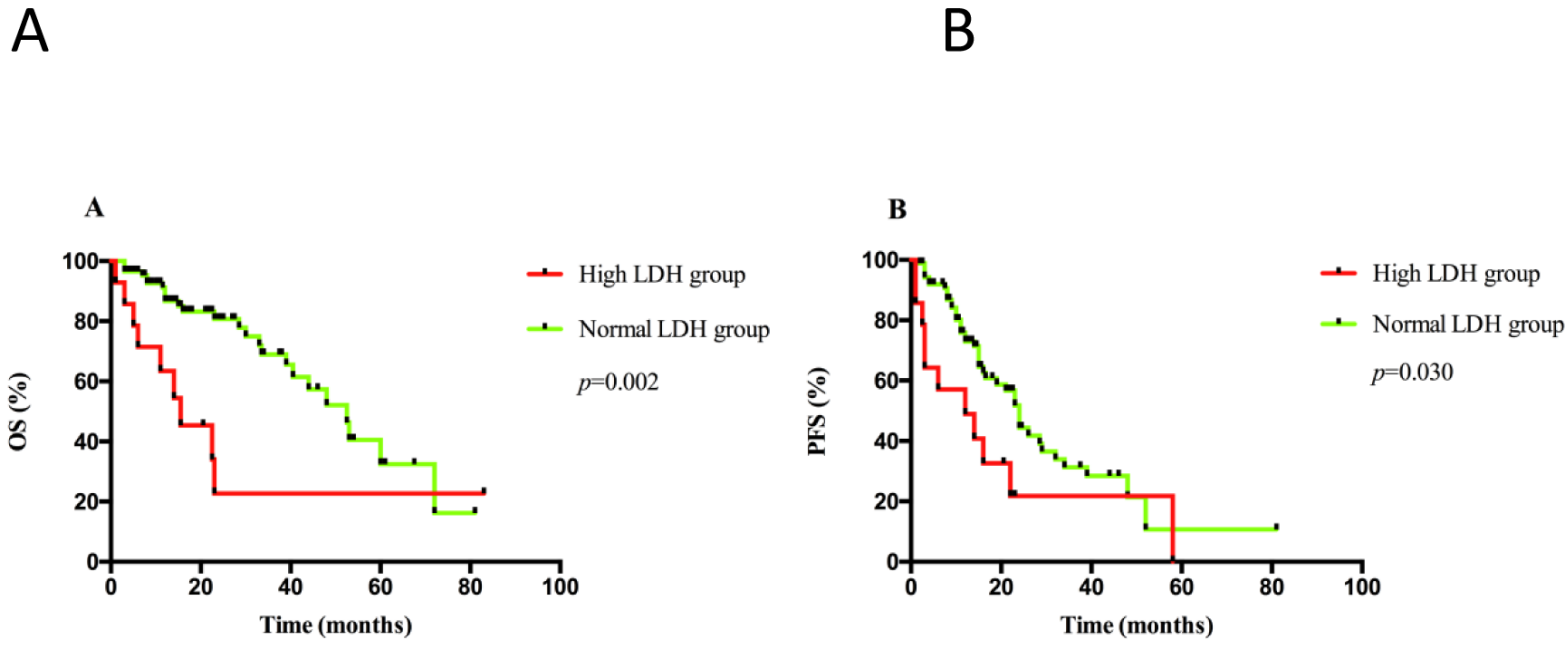
A. OS and B. PFS analyses were performed according to serum LDH levels at diagnosis in the whole cohort

The upper limit of normal LDH in our center is 271U/L. A total of 13.3% (14/105) patients had a high LDH level at diagnosis. With the median follow-up time of 15.5 (range, 1-83) months, median OS was 52.5 months (95% CI: 42.0-62.5) in normal LDH group, whereas 15.5 months (95% CI: 4.5-26.5) in elevated LDH group (*p* = 0.002). Medial PFS was 24.0 months (95% CI: 21.0-27.0) in normal LDH group, while 12.0 months (95% CI: 1.0-25.0) in high LDH group (*p* = 0.030) (Figure 1). The 2-year OS and PFS rate was 81% *versus* 20% (*p* < 0.001) and 44% *versus* 22% (*p* = 0.005), respectively.

### Univariate and multivariate cox regression analyses for survival in elderly patients with MM

In the univariable Cox analysis, the risk of death was increased for high LDH *versus* normal LDH (HR 3.208, 95% CI 1.470-6.998, *p* = 0.003), as well as high CRP *versus* normal CRP (HR 4.302, 95% CI 1.685-10.986, *p* = 0.002), whereas decreased for normal HB *versus* low HB (HR 0.344, 95% CI 0.133-0.891, *p* = 0.028). Male sex group demonstrated the trend for separating the two K-M curves with the *p*-value < 0.020 (HR 1.807, 95% CI 0.856-3.814, *p* = 0.121, Table 2). On the other hand, the risk of progression was increased for high LDH *versus* normal LDH (HR 2.076, 95% CI 1.053-4.093, *p* = 0.035), as well as high CRP *versus* normal CRP (HR 2.339, 95% CI 1.202-4.553, *p* = 0.012), while decreased for normal HB *versus* low HB (HR 0.470, 95% CI 0.241-0.916, *p* = 0.028). Erythrocyte sedimentation rate (ESR) demonstrated the trend for separating the two K-M curves with the *p*-value of which was < 0.020 (HR 2.402, 95% CI 0.572-10.085, *p* = 0.199, Table 3).

**Table 2:** Univariate analyses for overall survival in elderly patients with MM

Variables	Patients (n,%)	Median OS (months)	HR (95% CI)	*p*
Gender Female Male Age <70yr ≥70yr LDH ≤271U/L >271U/L ALB <35g/L ≥35g/L HB ≤100g/L >100g/L BPC ≤ 100×10^9^/L >100×10^9^/L β2-MG <3.5mg/L ≥3.5mg/L CRP <6mg/L ≥6mg/L FER <322ug/L ≥322ug/L ESR <20mm/H ≥20mm/H BUN <8.2mmol/L ≥8.2mmol/L sCr <2mg/dL ≥2mg/dL PCs <30% ≥30%	43 (41.0) 62 (59.0) 57 (54.3) 48 (45.7) 91 (86.7) 14 (13.3) 74 (70.5) 31 (29.5) 77 (73.3) 28 (26.7) 27 (25.7) 78 (74.3) 19 (18.1) 86 (81.9) n=77 45 (58.4) 32 (41.6) n=77 35 (45.5) 42 (54.5) n=78 7 (9.0) 71(91.0) n=104 72 (69.2) 32 (30.8) 76 (72.4) 29 (27.6) n=99 58 (58.6) 41 (41.4)	60.0 40.5 53.0 44.0 52.5 12.5 52.5 33.0 40.5 60.0 48.0 44.0 60.0 40.5 NR* 33.0 48.0 40.5 72.0 39.0 44.0 48.0 48.0 44.0 52.5 40.5	1.807 (0.856-3.814) 1.236 (0.620-2.466) 3.208 (1.470-6.998) 1.480 (0.661-3.315) 0.344 (0.133-0.891) 0.764 (0.361-1.617) 1.458 (0.562-3.787) 4.302(1.685-10.986) 1.313 (0.510-3.381) 1.600 (0.371-6.901) 0.909 (0.422-1.960) 0.978 (0.455-2.102) 1.348 (0.672-2.704)	0.121 0.547 0.003 0.340 0.028 0.482 0.439 0.002 0.573 0.528 0.808 0.954 0.400

*NR: Not Reached

**Table 3:** Univariate analyses for progression-free survival in elderly patients with MM

Variables	Patients (n,%)	Median PFS (months)	HR (95% CI)	p
Gender Female Male Age <70yr ≥70yr LDH ≤271U/L >271U/L ALB <35g/L ≥35g/L HB ≤100g/L >100g/L BPC ≤ 100×10^9^/L >100×10^9^/L β2-MG <3.5mg/L ≥3.5mg/L CRP <6mg/L ≥6mg/L FER <322ug/L ≥322ug/L ESR <20mm/H ≥20mm/H BUN <8.2mmol/L ≥8.2mmol/L sCr <2mg/dL ≥2mg/dL PCs <30% ≥30%	43 (41.0) 62 (59.0) 57 (54.3) 48 (45.7) 91 (86.7) 14 (13.3) 74 (70.5) 31 (29.5) 77 (73.3) 28 (26.7) 27 (25.7) 78 (74.3) 19 (18.1) 86 (81.9) n=77 45 (58.4) 32 (41.6) n=77 35 (45.5) 42 (54.5) n=78 7 (9.0) 71(91.0) n=104 72 (69.2) 32 (30.8) 76 (72.4) 29 (27.6) n=99 58 (58.6) 41 (41.4)	24.0 22.0 24.0 22.0 24.0 12.0 23.0 24.0 19.0 24.0 26.0 22.0 24.0 22.0 39.0 16.0 22.0 23.0 52.0 22.0 23.0 24.0 23.0 24.0 23.0 23.0	1.331 (0.751-2.360) 1.036 (0.601-1.783) 2.076 (1.053-4.093) 1.146 (0.613-2.145) 0.470 (0.241-0.916) 1.189 (0.623-2.269) 1.284 (0.625-2.635) 2.339 (1.202-4.553) 1.004 (0.502-2.006) 2.402 (0.572-10.085) 1.085 (0.601-1.960) 1.067 (0.592-1.925) 1.120 (0.635-1.976)	0.327 0.900 0.035 0.670 0.026 0.599 0.496 0.012 0.992 0.199 0.787 0.828 0.696

In the multivariable Cox analysis, male sex, LDH ( ≥ 271U/L), HB ( ≥ 100g/L), CRP ( ≥ 6mg/L), and ESR ( ≥ 20mm/H) were included as mentioned above. The unique risk of death was increased for high LDH *versus* normal LDH (HR 4.925, 95% CI 1.376-17.634, *p* = 0.014), as well as progression (HR 3.264, 95% CI 1.156-9.271, *p* = 0.026, Table 4).

**Table 4:** Multivariate Cox hazards regression analyses for survival in elderly patients with MM

Variables	OS			PFS		
	HR	95% CI	p	HR	95% CI	p
Gender (Male) LDH (>271U/L) HB (>100g/L) CRP (≥6mg/L) ESR(≥20mm/H)	1.657 4.735 0.603 3.226 2.391	0.370-7.427 1.420-15.793 0.127-2.853 0.791-13.153 0.281-20.320	0.509 0.011 0.523 0.102 0.425	1.866 3.513 0.540 1.631 3.784	0.625-5.571 1.243-9.927 0.172-1.692 0.581-4.576 0.490-29.270	0.264 0.018 0.290 0.353 0.202

**Table 5:** Univariate and multivariate logistic regression analyses for overall response rate in elderly patients with MM

Variables	Univariate analyze			Multivariate analyze		
	B	OR (95% CI)	p	B	OR (95% CI)	p
Gender (Male) Age (≥70yr) LDH (>271U/L) sCr (≥2mg/dL) ALB (≥35g/L) β2-MG (≥5.5mg/L) HB (>100g/L) BPC (>100×109/L) CRP (≥6mg/L) FER (≥20mm/H) BUN (≥8.2mmol/L) PCs (≥30%)	-0.760 0.177 1.898 0.629 -0.123 0.749 -0.413 0.025 0.217 0.339 0.384 -0.288	0.468 (0.187-1.170) 1.194 (0.483-2.950) 6.671(1.974-22.536) 1.875 (0.716-4.907) 0.884 (0.322-2.431) 2.097 (0.830-5.293) 0.661 (0.217-2.015) 1.026 (0.352-2.992) 1.243 (0.430-3.592) 1.403 (0.472-4.175) 1.469 (0.569-3.789) 0.750 (0.288-1.953)	0.104 0.702 0.002 0.200 0.812 0.117 0.467 0.963 0.688 0.543 0.427 0.556	-0.775 1.865 0.428 0.226	0.461 (0.170-1.246) 6.456 (1.803-23.120) 1.533 (0.448-5.251) 1.254(0.382-4.116)	0.127 0.004 0.496 0.709

**Table 6:** Univariate and multivariate logistic regression analyses for early death in elderly patients with MM

Variables	Univariate analyze			Multivariate analyze		
	B	OR (95% CI)	p	B	OR (95% CI)	p
Gender (Male) Age (≥70yr) LDH (>271U/L) sCr (≥2mg/dL) ALB (≥35g/L) β2-MG (≥5.5mg/L) HB (>100g/L) BPC (>100×109/L) CRP (≥6mg/L) FER (≥20mm/H) BUN (≥8.2mmol/L) PCs (≥30%)	1.435 -0.217 2.421 1.279 0.017 1.112 -19.938 -0.017 2.085 1.099 1.146 1.407	4.200 (0.484-36.441) 0.805 (0.170-3.816) 11.520 (2.165-58.551) 3.593 (0.747-17.303) 1.017 (0.185-5.601) 3.041 (0.557-16.586) - 0.984 (0.179-5.419) 8.043 (0.883-73.246) 3.000 (0.295-30.498) 3.147 (0.656-15.099) 4.083 (0.745-22.388)	0.193 0.785 0.004 0.111 0.985 0.199 0.998 0.985 0.064 0.353 0.152 0.105	1.385 3.347 -0.025 0.978 2.132 1.385	3.993 (0.190-84.086) 28.411(2.230-362.037) 0.975 (0.047-20.267) 2.659 (0.097-72.758) 8.431 (0.315-225.579) 3.993 (0.190-84.086)	0.373 0.010 0.987 0.562 0.204 0.232

### Comparison between ISS and R-ISS in survival in elderly patients with MM

**Figure 2:**
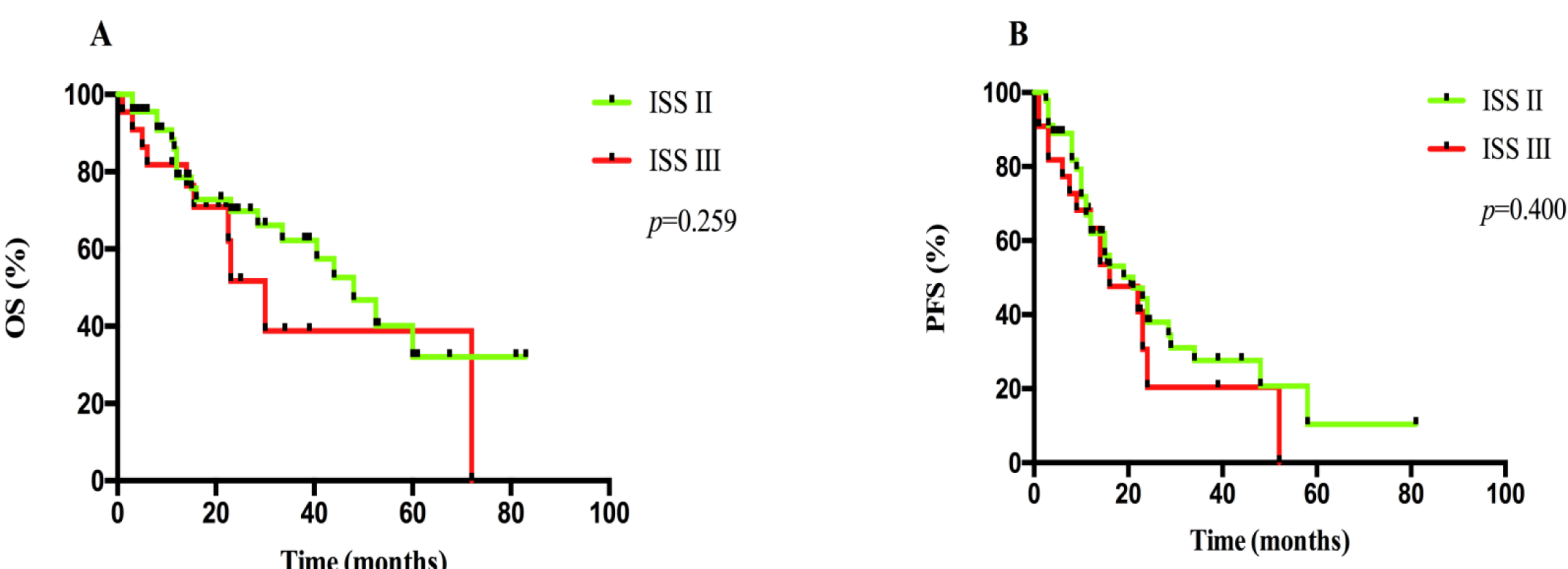
A. OS and B. PFS analyses between patients at ISS II and III

**Figure 3:**
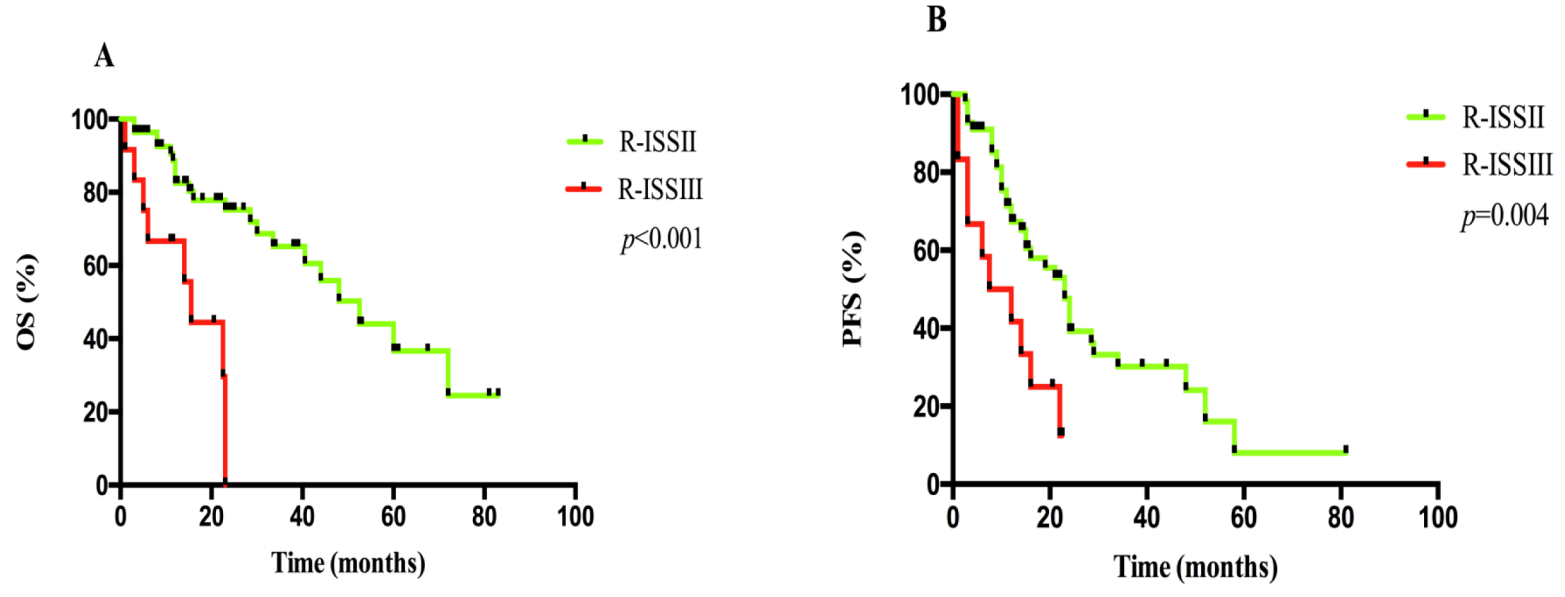
A. OS and B. PFS analyses between patients at R-ISS II and III

A total of 70 patients with complete LDH level, FISH and other clinical data were compared by ISS and R-ISS staging system. According to ISS, 1 case had stage I, 47 cases had stage II and 22 cases had stage III. Median OS in ISS II and III patients were 48.0 months (95% CI: 34.5-61.5) and 30.0 months (95% CI: 20.5-39.5), respectively (*p* = 0.259), while median PFS were 21.0 months (95% CI: 10.0-32.0) and 16.0 months (95% CI: 6.5-25.5), respectively (*p* = 0.400), as shown in Figure 2. The 2-year OS rates were 70% *versus* 51% (*p* = 0.368), whereas the 2-year PFS rates were 38% *versus* 20% (*p* = 0.425). We applied R-ISS by combination of LDH level and chromosomal abnormalities of t (4; 14), t (14; 16) and/or del (17p) with ISS: 1 case had stage I, 57 cases had stage II, and 12 cases had stage III. Median OS in R-ISS II and III patients were 52.5 months (95% CI: 38.0-67.0) and 15.5 months (95% CI: 11.5-19.5), respectively (*p* < 0.001), while median PFS were 23.0 months (95% CI: 18.5-27.5) and 7.5 months (95% CI: 1.0-17.5) respectively (*p* = 0.004), as shown in Figure 3. The 2-year OS rate was 75% *versus* 0% (*p* = 0.003), whereas the 2-year PFS rate was 39% *versus* 0% (*p* = 0.007).

### Univariate and multivariate logistic analyses for overall response rate in elderly patients with MM

Of all 105 patients, 4 cases refused any treatment, while another 7 cases lost to follow-up. According to the IMWG criteria, 72.3% (68/94) of patients achieved a partial response or better, while 27.7% (26/94) achieved less than partial response. ORR in high LDH group was 35.7% (5/14), compared to 78.8% (63/80) in normal LDH group (*p* = 0.002).

In the univariate analysis, the risk of not achieving PR was increased for patients who had high LDH *versus* normal LDH (OR 6.671, 95% CI 1.947-22.536, *p* = 0.002). In multivariate logistic regression analysis, male sex, sCr ≥ 2mg/dL, β2-MG ≥ 5.5mg/L were included because the p-value of them were < 0.020, as well as LDH ≥ 271U/L. Similarly, LDH was the unique independent negative factor for ORR in elderly patients as the risk of not achieving PR was increased for patients with high LDH *versus* normal LDH (OR 6.456, 95% CI 1.803-23.120, *p* = 0.004).

### Univariate and multivariate logistic analyses for early death in elderly patients with MM

Within the 94 patients who were followed-up over 6 months, early death occurred in 7.4% (7/94) of patients. The incidence in high LDH group was 28.6% (4/14), which was significantly higher compared with patients with normal LDH levels (3.75%, 3/80, *p* = 0.007). In univariate analysis, the risk of early death was increased for patients who had high LDH *versus* normal LDH (OR 11.520, 95% CI 2.165-58.551, *p* = 0.004). In multivariate logistic regression analysis, male sex, sCr ≥ 2mg/dL, β2-MG ≥ 5.5mg/L, CRP ≥ 6mg/L and PCs ≥ 30% were included because the *p*-value of them were < 0.020, as well as LDH ≥ 271U/L; while BUN was excluded because of the collinearity with sCr. Similarly, LDH was the unique independent adverse factor for early death in elderly NDMM patients as the risk of early death increased for patients with high LDH *versus* normal LDH (OR 28.411, 95% CI 2.230-362.037, *p* = 0.010).

## DISCUSSION

It has been reported that increased LDH is correlated with malignant degree and inferior prognosis. In addition, many studies have indicated that LDH was one of the variables for poor outcome in patients with MM, as well as advanced stage, chromosomal abnormalities, high serum free light chain and other factors [10, 11]. However, there was limited evidence in the elderly patient. In our study, patients in high LDH group had a median OS of 15.5 months and 2-year OS rate of 20%, both nearly a quarter of the normal LDH group; while median PFS and 2-year PFS rate were both approximately half of the normal LDH group. Compared to the relative younger patients, the impact of increased LDH was stronger in elderly patients more than 65 years old.

With the application of novel agents, overall survival of MM has improved in recent decades. However, increases were much less pronounced in patients aged 60 to 69 years [9], and no improvement was seen in even older patients [12–14]. It was partly because of the patients-specific factors such as age greater than 70 years, frailty, and comorbidities such as renal failure, cardiac failure [9, 15], but more important were the so-called disease-specific factors. As the results mentioned before, elderly patients in high LDH group demonstrated significant shorter survival compared with those had normal LDH level. Since more than 90% patients received novel agents in our study, such results confirmed the important prognostic predictive value of serum LDH for the novel agent efficacy in elderly MM patients.

To achieve a better understanding of the factors causing such poor survival, we analyzed several factors including LDH levels and their prognostic values. High LDH, elevated CRP and low HB levels were correlated with both shorter OS and PFS according to univariable analysis. Unlike other reports, multivariate analysis in our study showed LDH a unique independent predictor for both prior OS and PFS. Notably, although both BPC and β2-MG levels were correlated with LDH level in our cohort, they were no association with poor survival, possibly due to the subset of Asian elderly patients, the treatment of novel agents and adequate supportive care. Our results provided evidence that LDH is superior to other indicators such as HB, β2-MG, BPC, sCr and PCs for prognosis assessment in elderly MM patients, and can be used as an important indicator for prognostic prediction.

Based on a multi-center study enrolling 11751 patients with symptomatic MM, Greipp et al. have reported that β2-MG and serum albumin are most closely correlated with the prognosis by the multivariate analysis, and on this basis the International Staging System (ISS) was developed [16]. This system overcomed several limitations of Durie-Salmon staging system, which was proposed by Professor Durie in 1975 [17]. Although worldwidely applied for many years, ISS was developed prior to the introduction of novel agents for MM in the recent decades. More importantly, according to the results by Durie and IMWG, there were no significant differences in survivals between ISS II and III in Asian MM patients [16]. In addition, karyotype abnormalities have showed increasing prognostic value, and a growing number of evidences have indicated that molecular and cytogenetic heterogeneity may serve as clinical prognostic markers for MM [10, 18, 19]. At present, CD138 magnetic bead separation combined with FISH technology have considerably improved the cytogenetic abnormalities detection of MM [20–21] Given these therapeutic innovation and technical improvement, R-ISS was proposed by IMWG in 2015 which combines ISS, LDH and high-risk cytogenetic abnormalities including t (4;14), t (14,16) and/or del (17p) [22]. By applying the 70 elderly NDMM patients according to both ISS and R-ISS, we found that the amount of patients at R-ISS II significantly increased and an even worse prognosis in R-ISS III patients, while the survivals between the 2 stages in ISS system showed no differences. This finding demonstrated that R-ISS was a better staging system for prognostic prediction in elderly MM patients.

In addition to survivals, we also evaluated other prognostic parameters and their correlations with LDH levels. Of all the patients, normal LDH group exhibited two-fold or higher ORR compare with high LDH group. Both univariate and multivariate logistic regression analysis indicated that high LDH level was the unique independent indicator for not achieving partial response effects or better, even in the current era of novel agents. Compared to the younger patients, early mortality remains problematic in elderly patients with MM. Barlogie et al [7] suggested that high level of serum LDH and calcium decreased the early survival of MM patients. Chen et al [27] reported that high β2-MG, high serum LDH, and low serum albumin levels were poor prognostic factors for early mortality. In our study for the elderly patients, both univariable and multivariable logistic regression analysis demonstrated that LDH was the unique independent adverse factor for early death as the risk of early death was over ten-fold for elderly patients with high LDH levels compared with those with normal LDH.

Although the current patient cohort was relatively small, our results demonstrated that elevated LDH was an important adverse prognostic factor in Chinese elderly NDMM patients. Taking into account that quantitative detection of LDH is a convenient, readily available and inexpensive method, it has been widely applied at the time of diagnosis. Serum LDH levels at NDMM can provide critical information for risk stratification of elderly patients with MM in clinical practice. Thus, more intensive therapies or other innovative treatment approaches should be considered for patients who have increased LDH.

In conclusion, LDH is a crucial and economic unfavorable predictor of Chinese elderly patients with MM. In addition, R-ISS based on LDH and cytogenetics is superior to ISS for Chinese elderly NDMM patients in prognostic assessment.

## MATERIALS AND METHODS

### Patients and clinical features

We studied 105 patients with MM aged ≥ 65 years at diagnosis between July 2009 and July 2016 in our center. The median onset age was 69 years (range, 65-87), 62.9% (66/105) of whom were male sex. According to Durie-Salmon stages, 6 patients had stage I, 9 had stage II, and 90 had stage III. According to International staging system (ISS), 8 had stage I, 47 had stage II, and 50 had stage III. IgG isotype was present in 52.4% (55/105) of patients, whereas 25.7% (27/105) of them had IgA, 1.9% (2/105) had IgD, 1.9% (2/105) had biphenotype and 21.9% (23/105) had light chain only (5 cases of κ light chain and 18 cases of λ light chain). The median follow-up time in this study is 15.5 (range, 1-83) months.

The selection of regime for primary induction therapy was based on patients’ characteristic, the risk of toxicity, the capacity of patients to tolerate treatment, and also the patients’ intension. Most of the patients (90.5%) received novel agents based therapy as the first-line treatment, including 42.9% (45/105) thalidomide based therapy, 35.2% (37/105) bortezomib based regime, and 12.4% (13/105) lenalidomide based therapy. For rest of the patients, 5.7% (6/105) received conventional chemotherapy such as melphanlan-predisone (MP) and vincristine-adriamycin- dexamethasone (VAD), whereas 4 patients refused any therapy.

### Serum LDH test

Serum LDH concentrations were detected by AU5400 automatic biochemical analyzer (Olympus, Japan), equipped with commercially available original kit reagent. Serum samples were collected and LDH levels were determined before the initiation of treatment in all patients. According to the upper limit of normal range in our center, high LDH was defined as a serum level greater than 271U/L, whereas normal LDH was defined less than it. All samples were collected with informed consent in accordance with the Declaration of Helsinki.

### Fluorescence *in situ* hybridization analyses

Three sites of genetic abnormalities of t (4; 14), t (14; 16) and del (17p) were detected by interphase fluorescence *in situ* hybridization (iFISH) analyses on the bone marrow plasma cells that were sorted by CD138 labeled magnetic bead. A total of 100 to 300 interphase nuclei from each sample were scored, as appropriate. Positive cutoff threshold was defined as more than 20% cells with abnormal signal in del (17p) and/or > 10% abnormalities in immunoglobulin H (IgH) translocations.

### Diagnostic criteria and outcome assessment

Myeloma was diagnosed using standard criteria [23, 24] and response to treatment was assessed using International Myeloma Working Group (IMWG) criteria [25, 26]. Survival was defined as time from diagnosis until death or last follow-up. PFS was defined as interval from the diagnosis to first progression, death, or last follow-up. Overall response rate (ORR) refers to achieve the response ≥ partial response (PR). Early mortality was defined as death by any cause within the first 180 days after pathological diagnosis [27].

### Statistical analysis

Summary statistics of patients’ clinical characteristics at diagnosis were expressed in terms of the median plus the distribution range, as they were not normally distributed in both groups. Non-parametric Mann-Whitney U test was used for comparison such continuous variables between subgroups according to LDH levels. Survival curves were plotted using Kaplan-Meier method and log-rank test was applied for comparison. According to K-M curves and univariable analysis, all variables with clearly separated patterns and a *p*-value of less than 0.20 were entered into multivariable analysis, which was performed by multivariate cox proportional hazards regression model. Univariable and multivariable analysis of ORR and early death were carried out by logistic regression model. Similarly, independent variables were firstly tested separately using a univariable analysis and were entered into multivariable analyses with a level of *p*-value < 0.20. All reported *p*-values were two-sided and confidence intervals refer to 95% boundaries. A level of *p*-value < 0.05 was considered to be statistically significant. All statistical analyses were performed using GraphPad Prism 6 (GraphPad Software, San Diego, CA) and SPSS (version 21.0) software (IBM Corporation, Armonk, NY, USA).
